# Genomic Characterization of Mutli-Drug Resistant *Pseudomonas aeruginosa* Clinical Isolates: Evaluation and Determination of Ceftolozane/Tazobactam Activity and Resistance Mechanisms

**DOI:** 10.3389/fcimb.2022.922976

**Published:** 2022-06-15

**Authors:** Ibrahim Bitar, Tamara Salloum, Georgi Merhi, Jaroslav Hrabak, George F. Araj, Sima Tokajian

**Affiliations:** ^1^ Department of Microbiology, Faculty of Medicine, University Hospital Pilsen, Charles University, Pilsen, Czechia; ^2^ Biomedical Center, Faculty of Medicine, Charles University, Pilsen, Czechia; ^3^ Department of Natural Sciences, School of Arts and Sciences, Lebanese American University, Byblos, Lebanon; ^4^ Department of Pathology and Laboratory Medicine, American University of Beirut Medical Center, Beirut, Lebanon

**Keywords:** ceftolozane/tazobactam (C/T), *Pseudomonas aeruginosa*, AmpC, porins, beta lactamases

## Abstract

Resistance to ceftolozane/tazobactam (C/T) in *Pseudomonas aeruginosa* is a health concern. In this study, we conducted a whole-genome-based molecular characterization to correlate resistance patterns and β-lactamases with C/T resistance among multi-drug resistant *P. aeruginosa* clinical isolates. Resistance profiles for 25 *P. aeruginosa* clinical isolates were examined using disk diffusion assay. Minimal inhibitory concentrations (MIC) for C/T were determined by broth microdilution. Whole-genome sequencing was used to check for antimicrobial resistance determinants and reveal their genetic context. The clonal relatedness was evaluated using MLST, PFGE, and serotyping. All the isolates were resistant to C/T. At least two β-lactamases were detected in each with the *bla*_OXA-4_, *bla*_OXA-10_, *bla*_OXA-50_, and *bla*_OXA-395_ being the most common. *bla*_IMP-15_, *bla*_NDM-1,_ or *bla*_VIM-2_, metallo-β-lactamases, were associated with C/T MIC >256 μg/mL. Eight AmpC variants were identified, and PDC-3 was the most common. We also determined the clonal relatedness of the isolates and showed that they grouped into 11 sequence types (STs) some corresponding to widespread clonal complexes (ST111, ST233, and ST357). C/T resistance was likely driven by the acquired OXA β-lactamases such as OXA-10, and OXA-50, ESBLs GES-1, GES-15, and VEB-1, and metallo- β-lactamases IMP-15, NDM-1, and VIM-2. Collectively, our results revealed C/T resistance determinants and patterns in multi-drug resistant *P. aeruginosa* clinical isolates. Surveillance programs should be implemented and maintained to better track and define resistance mechanisms and how they accumulate and interact.

## Introduction

Antimicrobial resistance has been a soaring global health care tolling problem (Tacconelli et al., 2018; Moghnieh et al., 2019). *Pseudomonas aeruginosa* is one of the leading multidrug resistant (MDR) nosocomial pathogens worldwide and defined by the World Health Organization as a critical health concern with limited effective treatment options and is associated with poor clinical outcomes (Weiner et al., 2016; Tacconelli et al., 2018). MDR *P. aeruginosa* isolates have a broad variety of mechanisms mediating antimicrobial resistance (Gellatly and Hancock, 2013; López-Causapé et al., 2018). These include the up-regulation of efflux pumps, loss of outer membrane porins, the production of AmpC, extended-spectrum β-lactamases (ESBLs) and carbapenemases, and modification of antimicrobial target sites (Lister et al., 2009; Zavascki et al., 2010; Bassetti et al., 2019). The overproduction of AmpC β-lactamase was also linked to cephalosporins resistance and which was not reversed by β-lactamase inhibitors such as tazobactam and clavulanic acid (Sligl et al., 2015).

Two drug combinations were developed to treat infections caused by resistant Gram-negative bacteria, namely ceftazidime/avibactam and ceftolozane/tazobactam (C/T) (Sold under the brand name Zerbaxa) (Wright et al., 2017). Ceftolozane is a cephalosporin derivative of ceftazidime with an intrinsic broad activity and is not hydrolyzed by most broad-spectrum β-lactamases such as ESBLs and AmpCs (van Duin and Bonomo, 2016). Ceftolozane is particularly active against *P. aeruginosa* exhibiting AmpC efflux pumps overexpression (Moyá et al., 2012; Giacobbe et al., 2018), and has a heavier pyrazole substituent at the 3-position side chain instead of the lighter pyridium in ceftazidime, enhancing steric hindrance and interfering with AmpC hydrolytic activity (van Duin and Bonomo, 2016). The combination of ceftolozane and the β-lactamase inhibitor tazobactam proved to be active against many, but not all, ESBL-producing Gram-negative bacteria (Farrell et al., 2014). In particular, C/T combination was active against MDR and carbapenem-resistant *P. aeruginosa* (Giacobbe et al., 2018). The overall clinical success rate was reported to be 76.2% among MDR and extensively drug-resistant (XDR) *P. aeruginosa* (Maraolo et al., 2020). C/T drug combination was also more effective compared to polymyxins or aminoglycosides (Pogue et al., 2019). However, regional variations in C/T resistance within MDR *P. aeruginosa* were reported (Farrell et al., 2014; Sid Ahmed et al., 2019; O’Neall et al., 2020), and AmpC hyperproduction was a factor linked to C/T resistance in *P. aeruginosa* (Cabot et al., 2014). C/T was also less efficient against ESBL producing *Escherichia coli* and *P. aeruginosa* (Ortiz de la Rosa et al., 2019). The C/T activity against the Gram-negative ESBL producers, including *E*. *coli*, *Klebsiella pneumoniae*, and *P. aeruginosa*, recovered from clinical settings in Lebanon was recently tested by Araj et al. (2020), and which revealed that the C/T against MDR *E*. *coli* and *K*. *pneumoniae* isolates were much lower (Araj et al., 2020). In light of the rapid increase in the number of C/T *P. aeruginosa* resistant isolates we thought of studying C/T activity against MDR *P. aeruginosa*, determining the susceptibilities of the isolates, and conducting a genome-based molecular characterization.

## Materials and Methods

### Ethical Approval

Ethical approval was not required. The isolates were collected as part of routine clinical care and patient data collection. No additional isolates were collected beyond those obtained from routine clinical care, and no diagnostic or treatment decisions were affected by the outcomes of this study.

### Bacterial Isolates and Identification

A total of 25 *P. aeruginosa* isolates were collected and identified by the Matrix-Assisted Laser Desorption/Ionization Time of Flight (MALDI-TOF) system (Bruker Daltonik, GmbH, Bremen, Germany) at the clinical microbiology laboratory of the American University of Beirut Medical Center (AUBMC), Beirut, Lebanon between December 2017 and November 2018. AUBMC is around 350-bed tertiary care major hospital in the country. The isolates were designated as ZBX-P1 to ZBX-P25.

### Antimicrobial Susceptibility Tests

Antimicrobial susceptibility was tested against 10 antibiotics including C/T, amikacin, aztreonam, cefepime, ceftazidime, ciprofloxacin, gentamicin, imipenem, tazobactam and colistin. Data obtained were interpreted according to the CLSI guidelines (CLSI, 2022). For C/T breakpoints in *P. aeruginosa*, the established EUCAST (2022) (EUCAST, 2022) clinical zone diameter breakpoints were followed; S ≥ 24 mm and R < 24 mm and the MIC breakpoints of S ≤ 4 mg/L and R > 4 mg/L. For disk diffusion, the disk content was set at CXA-TAZ 30-10 µg, by both EUCAST and CLSI (CLSI, 2022; EUCAST, 2022).

### Pulsed-Field Gel Electrophoresis (PFGE)

PFGE fingerprinting was performed using the SpeI restriction enzyme (ThermoScientific, Waltham, MA, USA), 1% SeaKem agarose gel, and the universal laboratory standard *Salmonella enterica subsp. enterica serovar Braenderup* (ATCC^®^ BAA664™) digested with XbaI restriction enzyme according to the standard PulseNet protocol (http://www.pulsenetinternational.org). Electrophoresis was performed using the Bio-Rad laboratories CHEF DR-III system (Bio-Rad Laboratories, Bio-Rad Laboratories Inc., Hercules, CA, USA) with a run time of 16 h and switch time of 5–40 s (https://www.cdc.gov/pulsenet/). Gels were stained with ethidium bromide. PFGE profiles were analyzed with the BioNumerics software version 7.6.1 (Applied Maths, Sint-Martens-Latem, Belgium), with banding patterns showing a difference in three or more bands being placed under distinct pulsotypes (Tenover et al., 1995). Pulsotypes were clustered using the BioNumerics software version 7.6.1 (Applied Maths, Sint-Martens-Latem, Belgium) with an optimization of 0.5% and tolerance of 0.5%.

### Whole-Genome Sequencing

DNA extraction was performed using the NucleoSpin Microbial DNA kit (Macherey-Nagel, Germany) according to the manufacturer’s instructions followed by long- read sequencing of the isolates (ZBX-1 to ZBX-25). PacBio long-read sequencing on the Sequel I platform (Pacific Biosciences, CA, USA) was performed. Library preparation was according to the manufacturer’s instructions for microbial isolate multiplexing. G-tubes (Covaris, USA) were used for DNA shearing, and no size selection was performed. Resulting contigs were polished with Pilon, v1.23 (Walker et al., 2014), and the overlapping ends of chromosomes were trimmed after manual inspection of reads mapped by BWA-MEM algorithm as implemented in BWA, v0.7.17 (Li and Durbin, 2010), and Bowtie, v2.3.4.2 (Langmead and Salzberg, 2012). Genome assembly was done using HGAP4 with minimum seed coverage of 30x (Chin et al., 2013).

### Genome Analysis

Genomes were annotated using the RAST server (http://rast.nmpdr.org) (Aziz et al., 2012). Resfinder (Zankari et al., 2012), the Comprehensive Antibiotic Resistance Database CARD v3.1.0 (Alcock et al., 2020), MLST v1.8 (Larsen et al., 2012), ISfinder database (Siguier et al., 2006), PlasmidFinder (Carattoli et al., 2014) and BLASTn (Wheeler et al., 2003) were used to identify resistance genes, sequence types (STs), ISs, and plasmid incompatibility group identification and manual curation of annotations, respectively. The serotypes of the isolates were determined using the PAst v1.0. (Thrane et al., 2016) PAst types were split into 12 serogroups covering the 20 serotypes (Thrane et al., 2016). Mutations in AmpC, AmpR, and other target proteins were examined by aligning and comparing the corresponding amino acid sequences to *P. aeruginosa* PAO1 reference genome (Accession no. **GCF_000006765.1**).

### Porin Analysis

Sequences of 27 genes encoding for porins were extracted from *P. aeruginosa* PAO1 reference genome (Accession no. **GCF_000006765.1**) available on the *Pseudomonas* Genome Database (https://pseudomonas.com/) (Winsor et al., 2016). These sequences were used to build a dataset in MyDbFinder 2.0 (https://cge.cbs.dtu.dk/services/MyDbFinder/). Genomes were blasted against the dataset using a 60% ID threshold and a 40% minimum length for comparison.

### Data Availability

The Whole Genome Shotgun project was deposited at DDBJ/ENA/GenBank under the accession numbers presented in **S1 Table**.

## Results

### Patient Data

The mean patients’ age was 62.3 ± 18.9 years old. 64% (n=16) were males and 36% (n=9) were females and were collected from various sites of infection including: deep tracheal aspirate (DTA) (32%; n=8), urine (32%; n=8), wound (12%; n=3), sputum (12%; n=3), fluids, bone, and blood (4%; n=1).

### Genome Statistics and Isolate Genotyping

**Figure 1:**
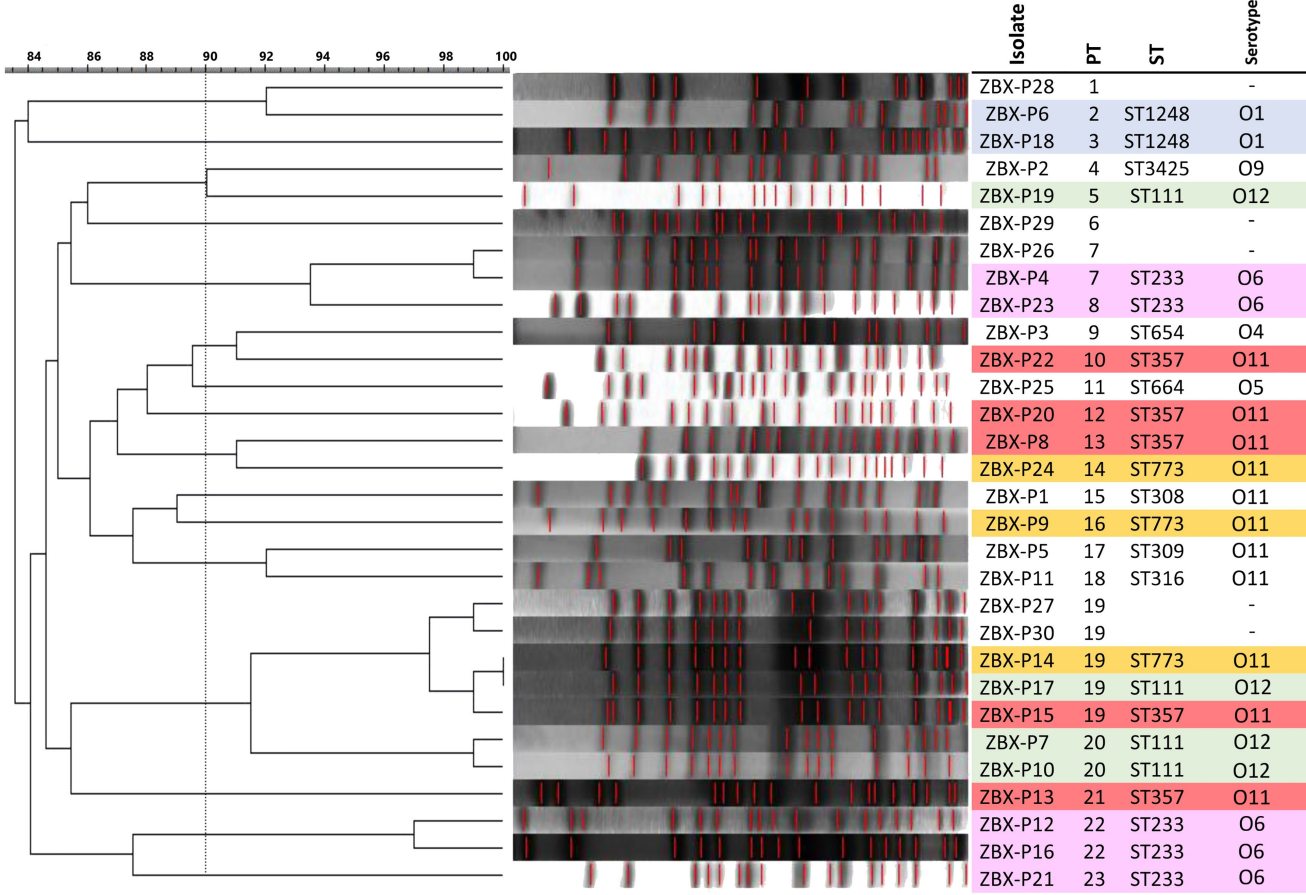
β-lactamases and AmpC antibiotic resistance genes identified in the isolates. Isolate’s ST is also shown.

The average genome size of the sequenced isolates was 6,972,377 ± 255,784 bp, and the average G+C content was 65.9 ± 0.2%. The clonal relatedness was evaluated using MLST, PFGE, and serotyping. The isolates were grouped into eleven STs (**Figure 1**) (one new: ST-3425). ST111 (n = 4), ST654 (n= 1), ST308 (n= 1), and ST357 (n= 4) are widespread “high-risk” clones (Woodford et al., 2011; Oliver et al., 2015; Papagiannitsis et al., 2017). The isolates were distributed within seven serotypes and 23 pulsotypes (PT1 to 23) showing ≥84% similarity based on PFGE (**Figure 1**).

### Antibiotic Susceptibilities

**Figure 2:**
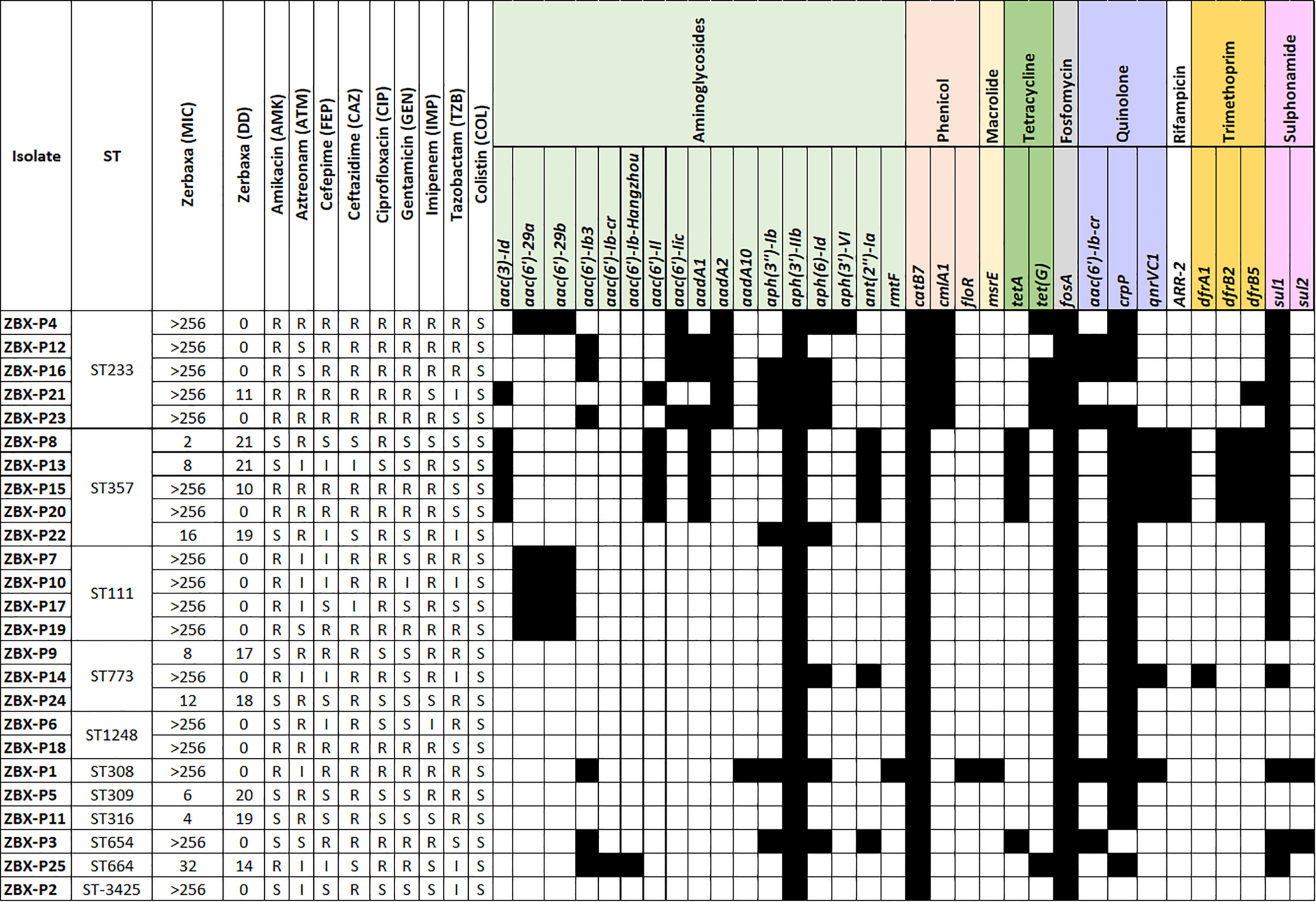
PFGE dendrogram and Serotype in the C/T *P. aeruginosa* isolates. Dendogram was generated by BioNumerics software version 7.6.1 showing the relationship of the isolates based on their banding patterns generated by SpeI restriction digestion. Isolates’ STs, and pulsotypes (PT) are shown.

All the isolates were resistant to C/T with MICs ranging between 2 to >256 μg/mL, while most also showed resistance to the other tested β-lactams including ceftazidime (88%; n=22) and imipenem (68%; n=17) (**Figure 2**). All the isolates were susceptible to colistin, followed by ciprofloxacin (68%; n=17), gentamicin (n = 13; 52%) and amikacin (n = 11; 44%) (**Figure 2**).

### Resistance Genomics

**Figure 3:**
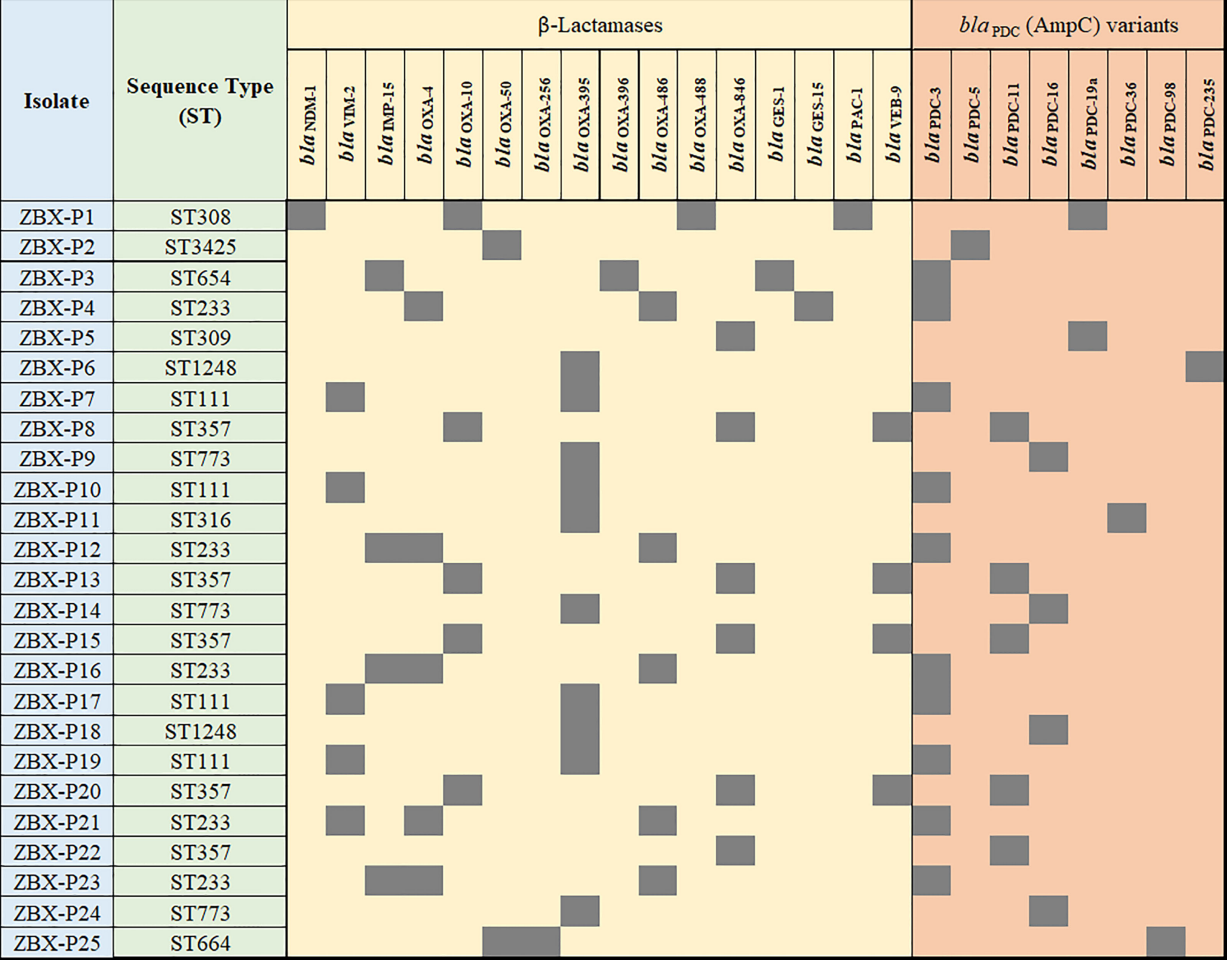
Detailed phenotypic and genotypic resistance information of the C/T resistant *P. aeruginosa* isolates. ST, Sequence type. Classes of antibiotic resistance genes are marked as follows: aminoglycoside, phenicol, macrolide, tetracycline, fosfomycin, quinolone, rifampicin, trimethoprim, and sulphonamide resistance genes. MIC, Minimum inhibitory concentration (µg/ml), DD, Diffusion diameter (mm). Black, present.

Whole-genome sequencing was used to check for antimicrobial resistance determinants and reveal their genetic context (**Figure 3** and **Table 1**). Between two to four β-lactamases were detected in the sequenced genomes; all were chromosomal except for *bla*_GES-15_ (**Table 1**). *bla*_IMP-15_, *bla*_NDM-1_, or *bla*_VIM-2_, metallo-β-lactamases, were associated with C/T MIC >256 μg/mL. Other β-lactamases were also detected with the most common being *bla*_OXA-395_, *bla*_OXA-50_, *bla*_OXA-4_, and *bla*_OXA-10_, respectively (**Figure 3**). MIC distributions are shown in (**Figure 2**). All C/T resistant isolates with MIC > 256 were either intermediate (n =1) or resistant to ceftazidime (n = 15). Of these, nine had four β-lactamases with one being a metallo-β-lactamase.

**Table 1:** Genetic environment of detected β-lactamases.

β-lactamases	Location		Isolates	ST	MGE	Closest *P. aeruginosa* Strain* (Accession #)	Reference
*bla*_GES-1_	C	I	ZBX-P3	ST-654	Tn*5393C*	PA34 (MF487840)	(Subedi et al., 2018)
*bla*_GES-15_	P		ZBX-P4	ST-233	IncP6 plasmid		
*bla*_IMP-15_	C	I	ZBX-P12, ZBX-P16, ZBX-P23	ST-233	Class I integron	1334/14 (CP035739.1)	(Garza-Ramos et al., 2010)
	C	I	ZBX-P3	ST-654	Class I integron	NCGM257 (AP014651.1)	
*bla*_NDM-1_	C	I	ZBX-P1	ST-308	ICE_Tn4371_ 6385	PASGNDM699 (CP020704.1)	(Ding et al., 2018)
*bla*_VIM-2_	C	I	ZBX-P21	ST-233	TniC-like class 1 integron	K34-7 (CP029707.1)	(Taiaroa et al., 2018)
	C	I	ZBX-P7, ZBX-P10, ZBX-P17, ZBX-P19	ST-111	Class 1 integron In58	RON-2 (AF263520.1)	(Poirel et al., 2001)
*bla*_VEB-1_	C	I	ZBX-P8, ZBX-P13, ZBX-P15, ZBX-P20	ST-357	Class I integron	GIMC5020:PA52Ts2 (CP051768.1)	(Aubert et al., 2004)
*bla*_OXA-4_	C	I	ZBX-P4, ZBX-P12, ZBX-P16, ZBX-P21, ZBX-P23	ST-233	Class I integron In*Pa5.1*	CDN118 (CP054591.1)	^(^ Strãut et al., 2018 ^)^
*bla*_OXA-10_	C	I	ZBX-P8, ZBX-P13, ZBX-P15, ZBX-P20	ST-357	Type 3 class I integron	AR_0443 (CP029147.1)	(Maurya et al., 2017)
	C	I	ZBX-P1	ST-308	Tn*1721*-like transposon	174313 (MK534438.1)	
*bla*_OXA-50_	C		ZBX-P2	ST-3425		GIMC5021:PA52Ts17 (CP051770.1)	
			ZBX-P8, ZBX-P13, ZBX-P15, ZBX-P20, ZBX-P22	ST-357		GIMC5021:PA52Ts17 (CP051770.1)	
			ZBX-P5	ST-309		PcyII-40 (LR739069.1)	
			ZBX-P25	ST-664		GIMC5021:PA52Ts17 (CP051770.1)	
			ZBX-P21	ST3425		GIMC5021:PA52Ts17 (CP051770.1)	
*bla*_OXA-256_	C	I	ZBX-P25	ST-664	Class I integron	97 (CP031449.2)	(Maurya et al., 2017)
bla_OXA-395_	C		ZBX-P7, ZBX-P10, ZBX-P17, ZBX-P19	ST-111		AG1 (CP045739.1)	
			ZBX-P9, ZBX-P14, ZBX-P24	ST-773		PSE6684 (CP053917.1)	
			ZBX-P6, ZBX-P18	ST-1248		PABL048 (CP039293.1)	
*bla*_OXA-396_	C		ZBX-P3	ST-654		N15-01092 (CP012901.1)	
*bla*_OXA-486_	C		ZBX-P4, ZBX-P12, ZBX-P16, ZBX-P21, ZBX-P23	ST-233		AR_0111 (CP032257.1)	
*bla*_OXA-488_	C		ZBX-P1	ST-308		WPB100 (CP031877.1)	

MGE, Mobile genetic elements**;** β-lactamases location, Integron (I), chromosome (C), plasmid (P). #, number. *Obtained from BLAST.

All the isolates were intrinsic AmpC producers showing eight *ampC*-type variants (PDC) (**Figure 3**). We checked for polymorphisms in the AmpR (*bla*_PDC_ transcriptional regulator) aligning against PA4109 in *P. aeruginosa* PAO1 reference strain. Several substitutions were detected including D135N in isolates with C/T MIC < 256 μg/mL (ZBX-P9 MIC: 8 μg/mL, ZBX-P11 (MIC: 4 μg/mL, and ZBX-P24 MIC: 12 μg/mL), and another restricted to the ones typed as ST111 (E287G C/T MIC > 256 μg/mL). We also looked for OprD polymorphisms and detected the characteristic mutation (Q142X) in ZBX-P12, ZBX-P16, and ZBX-P23 (all with C/T MIC > 256 μg/mL).

Furthermore, *bla*_NDM-1_ along with *floR* and *msr*(E) were on a chromosomal 74.2 kb integrative and conjugative element (ICE) ICE_Tn4371_ *6385. bla*_OXA-488_ was also chromosomal, while *bla*_VIM-2_, *bla*_OXA-4_, and *bla*_OXA-10_ were all detected on chromosomal class I integrons, and *bla*_GES-15_ on IncP-6 plasmid (**Table 1**).

Finally, we compared 27 porin encoding genes with that of *P. aeruginosa* PAO1. The highest variability was observed in *oprD*, *opdD* (PA4501 and PA1025), and *oprQ*. Deletions and pre-mature stop codons (truncations) were detected throughout OprD, and the characteristic OprD mutation (Q142X) associated with C/T resistance (Fraile-Ribot et al., 2018) was found in ZBX-P12 and ZBX-P16, and ZBX-P23 (C/T MIC > 256 μg/mL).

## Discussion

Choosing the appropriate antimicrobial agent to treat infections caused by resistant bacteria would significantly decrease infection-linked morbidity and mortality. C/T has increased activity against resistant *P. aeruginosa* isolates and is an important treatment option in institutions with high rates of pseudomonal infections (Humphries et al., 2017; Hirsch et al., 2020). The emergence of C/T resistance in MDR/XDR *P. aeruginosa* isolates, resistant to most β-lactams and having several resistance mechanisms, is very likely to happen. In this study, we used long-read whole-genome sequencing approach to study the resistance genomics and clonal relatedness of 25 C/T resistant *P. aeruginosa.* The isolates belonged to 11 STs and 23 pulsotypes, exhibited C/T MIC of 1.5 to > 256 μg/mL, showed resistance to the other tested β-lactams including ceftazidime (88%; n=22), ciprofloxacin (68%; n=17), and imipenem (68%; n=17). Moreover, isolates had two to four β-lactamases and 10 were positive for *bla*_IMP-15_, *bla*_NDM-1,_ or *bla*_VIM-2_, and were intrinsic AmpC producers.

PDC variants in this study were assigned according to substitutions in AmpC described by Rodríguez-Martínez et al. (2009) and accordingly we identified eight *ampC* variants, PDC-3 being the most common. T79A (T105A non-processed peptide) detected in PDC-3 (Rodríguez-Martínez et al., 2009), was previously found to be prevalent in carbapenem-resistant clinical isolates. Berrazeg et al. (2015), however, overexpressed PDC-3 (T79A) in porin OprD-negative strain to test if it would potentiate the AmpC action and revealed that T79A variation doesn’t broaden the substrate spectrum of AmpC (Berrazeg et al., 2015). They concluded that PDC common polymorphisms had negligible impact on AmpC activity while confirming that mutations occurring in specific regions of the substrate-binding pocket could enhance the catalytic efficiencies and as a result increase the hydrolytic activity of *P. aeruginosa* AmpC on cephalosporins including ceftolozane. On the other hand, Fernández-Esgueva et al. (2020) showed that C/T resistance was associated with AmpC mutations including a novel one (PDC-388; G183V) (Fernández-Esgueva et al., 2020) and in line with this Fraile-Ribot et al. (2018) also cloned AmpC variants (PDC-221, 222, and 223) in *ampC*-deficient derivative of PAO1 (Fraile-Ribot et al., 2018). The cloned AmpC variants showed increased ceftolozane/tazobactam and ceftazidime/avibactam MICs compared with wild type AmpC with the associated polymorphisms being located within the Ω loop and selected *in vitro* upon C/T exposure (Cabot et al., 2014; Haidar et al., 2017). We detected the characteristic OprD mutation (Q142X) associated with C/T resistance (Fraile-Ribot et al., 2018) in three of the study isolates, ZBX-P12, ZBX-P16, and ZBX-P23 (C/T MIC > 256 μg/mL).

Moreover, C/T resistance emergence was previously reported in isolates producing horizontally acquired β-lactamases such as OXA-10 and OXA-2 (Fraile-Ribot et al., 2018), and ESBLs (Ortiz de la Rosa et al., 2019). In general, C/T may be ineffective against isolates carrying carbapenemases including class A and class D (OXA) β-lactamases and it’s inactive against metallo-β-lactamases (Hirsch et al., 2020; Karlowsky et al., 2021). Extended-spectrum OXAs were also noted as infrequent cause of ceftolozane resistance (Livermore et al., 2009; Juan et al., 2010). Our results mainly agreed with C/T resistance being linked to horizontally acquired β-lactamases. We detected two to four β-lactamases in the sequenced genomes. In line with this, Sid Ahmad et al. (2019) reported a strong association between MDR *P. aeruginosa* isolates displaying MIC_256_ to C/T and the presence of OXA-10, VIM-2, and OXA-488 with the highest association frequency being with class C and D β-lactamases. We also had metallo-β-lactamases including *bla*_IMP-15_, *bla*_NDM-1,_ or *bla*_VIM-2_ and which were invariably (except for one isolate) linked to C/T MIC >256 μg/mL.

Infections caused by MDR *P. aeruginosa* could be treated with C/T, clinical studies evaluating optimal dosing and using combined therapy are recommended (Fraile-Ribot et al., 2018). Mutations occurring within the substrate-binding pocket could increase the hydrolytic activity of *P. aeruginosa* AmpC on cephalosporins including ceftolozane. Horizontally acquired β-lactamases, and intrinsic AmpC modifications are the main mechanisms leading to C/T resistance in MDR *P. aeruginosa.* Our data don’t support the accumulation of mutations leading to the overexpression or structural modification of AmpC and so it’s more likely that C/T resistance was driven by the acquired OXA β-lactamases such as OXA-10, OXA-50, ESBLs GES-1, GES-15 and VEB-1, and metallo-β-lactamases IMP-15, NDM-1, and VIM-2. Collectively, our results highlight the need to maintain active surveillance programs to better track and define resistance mechanisms and how they accumulate and interact.

## Data Availability Statement

The datasets presented in this study can be found in online repositories. The names of the repository/repositories and accession number(s) can be found in the article/**Supplementary Material**.

## Author Contributions

GA: Conceptualization, Validation, Writing – review and editing. ST: Conceptualization, Funding acquisition, Project administration, Supervision, Validation, Writing – review and editing. IB: Funding acquisition, Investigation, Methodology, Writing – review and editing. TS: Investigation, Methodology, Visualization, Writing – original draft, Writing – review and editing. GM: Investigation, Writing – review and editing. JH: Investigation, Methodology. All authors contributed to the article and approved the submitted version.

## Funding

This work was supported by Lebanese American University Strategic Research Review Committee Grant (SRRC-R-2019-38) and by the research project grants NU20J- 05-00033 provided by Czech Health Research Council, by the Charles University Research Fund PROGRES (project number Q39), and by the project Nr. CZ.02.1.01/0.0/0.0/16_019/0000787 “Fighting Infectious Diseases” provided by the Ministry of Education Youth and Sports of the Czech Republic. The funders had no role in study design, data collection and analysis, decision to publish, or preparation of the manuscript.

## Conflict of Interest

The authors declare that the research was conducted in the absence of any commercial or financial relationships that could be construed as a potential conflict of interest.

## Publisher’s Note

All claims expressed in this article are solely those of the authors and do not necessarily represent those of their affiliated organizations, or those of the publisher, the editors and the reviewers. Any product that may be evaluated in this article, or claim that may be made by its manufacturer, is not guaranteed or endorsed by the publisher.

## Supplementary Material

Supplementary Table 1 List of the isolates’ accession numbers.
